# The possible mechanisms linking chronic obstructive pulmonary disease and coronary atherosclerosis based on coronary computed tomography angiography and animal experiments

**DOI:** 10.3389/fphys.2026.1688832

**Published:** 2026-05-07

**Authors:** Jie Li, Kewu Huang

**Affiliations:** 1 Department of Pulmonary and Critical Care Medicine, Beijing Chao-Yang Hospital, Capital Medical University, Beijing, China; 2 Department of Pulmonary and Critical Care Medicine, Beijing Anzhen Hospital, Capital Medical University, Beijing, China; 3 Beijing Institute of Respiratory Medicine, Beijing, China

**Keywords:** chronic obstructive pulmonary disease, coronary atherosclerosis, coronary stenosis, Kelch-like ECH-associated protein 1-nuclear factor erythroid 2-related factor 2 pathway, oxidative stress

## Abstract

**Objective:**

The study sought to evaluate the characteristics of coronary atherosclerosis (CAS) in patients with chronic obstructive pulmonary disease (COPD) based on computed tomography angiography (CTA), explore its relationship with COPD, and discuss the mechanisms of KEAP1–NRF2-mediated oxidative stress.

**Methods:**

A total of 300 COPD patients undergoing CTA were divided into COPD alone (n = 165) and COPD + CAS (n = 135) groups based on the presence/absence of CAS, and 120 CAS patients were enrolled as controls. Clinical and laboratory data were collected and compared. Patients were further stratified regarding COPD severity (76 mild, 84 moderate, 74 severe, and 68 very severe cases). Univariate/multivariate logistic regression identified factors influencing COPD with CAS. *In vivo* validation of KEAP1–NRF2-mediated oxidative stress was performed for mechanistic interpretation using a COPD and CAS comorbidity model.

**Results:**

Compared with patients with CAS or COPD alone, significant differences in terms of clinical baseline data were observed in patients with COPD and CAS. COPD patients exhibited reduced KEAP1, superoxide dismutase, and catalase activities, along with upregulated NRF2, reactive oxygen species, and malondialdehyde. These alterations were more pronounced in patients with more severe COPD and concomitant CAS. KEAP1 and NRF2 were independent influencing factors of comorbid COPD and CAS. In the mouse model, activating the KEAP1–NRF2-mediated oxidative stress was associated with aggravated lung injury and enlarged atherosclerotic lesion areas.

**Conclusion:**

COPD severity is closely associated with the burden of CAS. Dysregulation of the KEAP1–NRF2-mediated oxidative stress may represent a shared biological feature linking COPD and CAS.

## Introduction

Chronic obstructive pulmonary disease (COPD) denotes a type of lung disease associated with a decline in pulmonary function over time, featured by persistent respiratory symptoms and progressive airflow obstruction that is not fully reversible (Hattab et al., 2016; Labaki and Rosenberg, 2020). COPD causes substantial morbidity and mortality and imposes a considerable healthcare burden globally on society (Christenson et al., 2022). One of the most prevalent comorbidities in COPD patients is coronary atherosclerosis (CAS); both COPD and CAS share common risk factors, namely, cigarette smoke and ageing. Specifically, exposure to cigarette smoke can elicit a chronic low-grade systemic inflammation that characterizes both COPD and CAS. The development of CAS in COPD patients can enhance the morbidity of COPD and increase hospitalization, mortality, and healthcare costs (Ghoorah et al., 2013; Roversi et al., 2014). In turn, COPD is also an independent risk factor for CAS, and its specific mechanism may be associated with chronic systemic inflammation, which generates oxidative stress in the lungs, causes intravascular dysfunction, and promotes the formation and rupture of atherosclerotic plaques, consequently initiating CAS (Rao et al., 2005; Liang et al., 2013; Li et al., 2022). Nevertheless, the exact mechanisms behind the observed association between COPD and CAS are not well known.

Transcription factor nuclear factor erythroid 2-related factor 2 (NRF2) is known to be a master regulator of expression of antioxidant response element-driven cytoprotective proteins; its activation confers protective effects on cells and tissues against oxidative stress-induced injury (Liu et al., 2019). Notably, NRF2 activation is mainly achieved through the interactions with its inhibitor Kelch-like ECH-associated protein 1 (KEAP1), and the KEAP1–NRF2 pathway represents a cellular defensive system against oxidative stress (Lee and Hu, 2020; Sykiotis, 2021). In support, cryptotanshinone, an active ingredient in *Salvia miltiorrhiza*, has been documented to alleviate lipopolysaccharide and cigarette smoke-driven COPD in mice by repressing the protein level of KEAP1, resulting in the activation of NRF2 (Song et al., 2023). Additionally, the uptake of oxidized low-density lipoprotein (ox-LDL) by macrophages can trigger the accumulation of lipids and formation of foam cells, which is a critical event in the development of atherosclerosis (Saki et al., 2024). Importantly, NRF2 activation decreases the serum level of ox-LDL, inhibits endoplasmic reticulum stress, and reduces reactive oxygen species (ROS) accumulation in the aorta and macrophage-derived foam cells, therefore stabilizing atherosclerotic plaques (Song et al., 2015). Therefore, targeting KEAP1–NRF2-mediated oxidative stress stress may represent a potential approach to ameliorate COPD combined with CAS. Coronary computed tomography angiography (CTA) is known as a non-invasive imaging tool to assess coronary artery disease by quantifying plaque burden, stenosis, and extent of coronary artery plaques (Ru et al., 2021). It is noteworthy that coronary CTA (CCTA) can be employed for the diagnosis of cardiovascular events in patients affected by COPD (Wang et al., 2025). In this regard, the current study enrolled COPD patients who underwent CCTA to assess the characteristics of CAS in COPD patients, explore the association between COPD and CAS, and elucidate the regulatory mechanism underlying oxidative stress mediated by the KEAP1–NRF2 pathway. At the same time, we conducted animal experiments to validate the relationship between oxidative stress mediated by the KEAP1–NRF2 pathway and COPD progression.

## Materials and methods

### Ethics statement

Animal experiments were carried out by referring to the protocols approved by the Animal Ethics Committee of Beijing Anzhen Hospital. This was a retrospective study using existing clinical data. The protocol was reviewed and approved by the Academic Ethics Committee of Beijing Anzhen Hospital (Approval No. IRB-2021128X) and conformed to the *Declaration of Helsinki*. The requirement for informed consent was waived due to the retrospective nature of the study and use of de-identified data.

### Study subjects

From January 2022 to June 2024, 321 consecutive patients with COPD who underwent CCTA at Beijing Chao-Yang Hospital were retrospectively reviewed. After applying exclusion criteria, 21 cases were excluded, and 300 cases were finally enrolled. This cohort consisted of 165 COPD cases (no coronary abnormalities) and 135 cases with COPD combined with CAS (COPD + CAS). Among the 135 patients in the COPD + CAS group, 42 had coronary artery stenosis of <50%, 58 had stenosis of 50%–70%, and 35 had stenosis of >70%. An additional 120 patients with CAS alone (without COPD) were included during the same period to serve as controls. According to the 2017 Global Initiative for Chronic Obstructive Lung Disease (GOLD) guidelines, the 300 enrolled patients with COPD were further stratified based on symptom scores and risk of acute exacerbation (severity of COPD) into four groups: the mild group (n = 76), the moderate group (n = 84), the severe group (n = 72), and the very severe group (n = 68).

### Inclusion and exclusion criteria

COPD patients were included if they were aged ≥40 years, met the diagnostic criteria for COPD [post-bronchodilator forced expiratory volume in 1 s/forced vital capacity (FEV1/FVC) < 0.7] (Agusti et al., 2023), and underwent CCTA examination due to suspected coronary heart disease (e.g., symptoms such as chest pain and dyspnea) or the presence of high-risk factors.

The exclusion criteria for COPD patients included a history of coronary intervention within 6 months, pregnant or lactating women, comorbid severe cardiovascular diseases (such as acute myocardial infarction, severe heart failure, and severe arrhythmia), renal insufficiency, and other comorbid respiratory diseases (such as interstitial lung disease and lung cancer) that may interfere with COPD assessment.

COPD severity classification (GOLD 2017) (Houben-Wilke et al., 2018) is as follows. Mild: COPD Assessment Test (CAT) score < 10, with ≤1 exacerbation within the past 12 months (without hospitalization). Moderate: CAT score ≥ 10, with ≤1 exacerbation within the past 12 months (without hospitalization). Severe: CAT score < 10, with ≥2 exacerbations within the past 12 months or ≥1 hospitalization. Very severe: CAT score ≥ 10, with ≥2 exacerbations within the past 12 months or ≥1 hospitalization. CAT is an eight-item unidimensional measure used to assess the health status impairment in patients with COPD. It evaluates cough, sputum production, chest tightness, dyspnea, activity limitation, confidence leaving home, insomnia, and energy levels. Each item is scored from 0 to 5, with a total score ranging from 0 to 40, where higher scores indicate a greater symptom burden. Modified Medical Research Council (mMRC) dyspnea scale (Rhee et al., 2015): Grade 0, dyspnea only with strenuous exercise; Grade 1, shortness of breath and difficulty breathing during brisk walking or walking up a slight hill; Grade 2, walks slower than peers because of dyspnea or needs to stop and rest during walking; Grade 3, stops to rest after walking approximately 100 m; and Grade 4, too breathless to leave the house or perform daily movements such as getting dressed or undressing.

The inclusion criteria for CAS patients included age ≥ 18 years, meeting the diagnostic criteria for CAS according to the 2024 European Society of Cardiology Guidelines for the management of chronic coronary syndromes (Vrints et al., 2024), undergoing CCTA examination, and no hospitalization for acute coronary syndrome or planned revascularization within the 3 months prior to enrollment.

The exclusion criteria for CAS patients included history of coronary intervention within the preceding 6 months, pregnancy or lactation, concomitant severe cardiovascular diseases (such as acute myocardial infarction, severe heart failure, and severe arrhythmias), renal insufficiency, anatomically unsuitable conditions (such as left main stenosis ≥ 50%, chronic total occlusion, severe calcification or tortuosity, and in-stent restenosis), severe cardiac dysfunction or shock, and history of coronary artery bypass grafting.

### Data collection

Clinical data and biochemical indicators of all subjects were collected, including age, body mass index (BMI), sex, history of diabetes, history of hyperlipidemia, history of hypertension, history of coronary heart disease, smoking history, smoking pack-years, CAT score, and mMRC score. Other laboratory parameters on admission were recorded, including triacylglycerol (TG), total cholesterol (TC), low-density lipoprotein cholesterol (LDL-C), high-density lipoprotein cholesterol (HDL-C), FEV1, FVC, FEV1/FVC, hemoglobin (Hb), red blood cells (RBC), lipid peroxide (LPO), glutathione peroxidase (GSH-Px), catalase, high-sensitivity C-reactive protein (hsCRP), and interleukin-6 (IL-6).

Peripheral venous blood samples were collected from all subjects in the morning after admission under fasting conditions. Blood samples were collected into non-anticoagulant tubes (for serum separation) and ethylenediaminetetraacetic acid (EDTA)-anticoagulant tubes [for peripheral blood mononuclear cell (PBMC) isolation]. The non-anticoagulated blood samples were allowed to clot at room temperature and then centrifuged at 3,000 × *g* for 10 min to separate the serum. The serum was aliquoted and stored at −80 °C for the subsequent detection of biochemical markers related to oxidative stress and inflammation. The EDTA-anticoagulated blood samples were used for PBMC isolation and subsequent mRNA quantitative analysis.

### CCTA image analysis

All CCTA images were retrieved from the Picture Archiving and Communication System (PACS) of our hospital and processed using dedicated CTA post-processing software Syngo.via (Version VB30, Siemens Healthineers, Germany) for post-processing and quantitative analysis. CTA images were reconstructed and evaluated on the Syngo.via platform following standard cardiovascular analysis protocols, including multiplanar reconstruction (MPR) and curved planar reconstruction (CPR), combined with volume rendering (VR) when necessary, to comprehensively visualize coronary luminal structures and plaque characteristics. Coronary plaque analysis was performed segment by segment along the major coronary artery branches [left anterior descending artery (LADA), left circumflex artery (LCx), and right coronary artery (RCA)]. Plaque segmentation was conducted using the semi-automated plaque analysis tool integrated into the Syngo.via software. Based on the automatic identification of vessel contours and plaque regions, manual corrections were applied by the readers when necessary to enhance segmentation accuracy and consistency. Quantitative plaque parameters included plaque type, plaque volume, plaque burden, and degree of coronary stenosis.

Qualitative analysis was performed on the plaques in the coronary arteries of patients with COPD. Based on the mean plaque Hounsfield units (HU), plaque type was categorized as calcified plaques with a CT density of >130 HU (high-density areas with clear boundaries) and non-calcified plaques with a CT density of <130 HU (low-density or equal-density areas with blurred boundaries). According to the Agatston coronary artery calcification score (CACS), patients were divided into mild (defined as CACS = 1–99), moderate (defined as CACS = 100–399), and severe (defined as CACS > 400) coronary calcification. CACS was conducted using an unenhanced low-dose CT scan (Tang et al., 2024). The scoring was based on the volume and density of coronary artery calcification under CT scan, with a higher score indicating higher levels of coronary calcification. A total calcification score of 0 refers to no plaque, a score of 1–99 refers to mild calcification, a score of 100–399 refers to moderate calcification, and a score greater than 400 refers to severe calcification. The degree of coronary artery stenosis was determined based on the most severe narrowing at the patient level: the maximum stenosis severity among the LADA, LCx, and RCA was graded, with <50% defined as mild, 50%–70% as moderate, and ≥70% as severe.

All CTA images were independently analyzed by two radiologists with experience in cardiovascular imaging diagnosis, who were blinded to the patients’ clinical data and group allocation. In cases of disagreement between the two readers regarding plaque characteristics or stenosis severity, a consensus was reached through joint review and discussion. All image analyses were performed on a unified software platform and using standardized operating procedures to minimize potential bias from subjective judgment.

### Experimental animals and treatment

A total of 36 male ApoE^−/−^ mice (8 weeks old; weighing 20 ± 2 g) and six male C57BL/6J wild-type (WT) mice (weighing 22 ± 2 g each) of the same genetic background and age were used in this study. The WT mice served as normal controls (Control). All mice were purchased from Beijing Vital River Laboratory Animal Technology Co., Ltd. (Beijing, China) and housed under specific pathogen-free conditions with an ambient temperature of 26 °C–28 °C, relative humidity of 50%–65%, and free access to food and water. All animals were allowed to acclimatize for 1 week prior to the experiment.

Mice were randomly divided into six groups (n = 6 per group) using a random number table. 1) Control group: WT mice were fed a standard diet without cigarette smoke exposure. 2) COPD group: ApoE^−/−^ mice were subjected to cigarette smoke exposure in glass chambers (twice daily, 1 h per session, 5 days per week for 10 consecutive weeks) and fed a standard rodent diet. 3) COPD + CAS group: ApoE^−/−^ mice were fed a high-fat, high-cholesterol diet (Wang et al., 2022) under the same cigarette smoke exposure conditions as the COPD group. 4) COPD + CAS + NAC group: based on COPD + CAS modeling, ApoE^−/−^ mice were given intraperitoneal injection of *N*-acetylcysteine (NAC; HY-153340, MedChem Express LLC, NJ, USA) at a dose of 100 mg/kg/day starting from the second week, eight consecutive weeks in total (Demiroren et al., 2018). 5) COPD + CAS + AAV-si-KEAP1 group: based on the COPD + CAS modeling, ApoE^−/−^ mice received a tail vein injection of a recombinant adeno-associated virus vector AAV-si-KEAP1 (3 × 10^11^ virus/200 μL phosphate-buffered saline) at the second week to knock down KEAP1 expression *in vivo* (Tong et al., 2023). 6) COPD + CAS + AAV-si-NC group: ApoE^−/−^ mice received a tail vein injection of an equivalent dose of negative control vector AAV-si-NC at the same time point. At the experimental endpoint, mice were anesthetized *via* intraperitoneal injection of 1% sodium pentobarbital (45 mg/kg) for a pulmonary function test (PFT). Subsequently, under deep anesthesia, terminal blood samples were harvested *via* cardiac puncture, and the mice were euthanized with an intraperitoneal injection of sodium pentobarbital (100 mg/kg). Whole lung tissues were harvested. The left lung tissue was processed to prepare tissue homogenates for biochemical and molecular analyses, while the right lung tissue was fixed, embedded in paraffin, and subjected to hematoxylin and eosin (H&E) staining to evaluate histopathological changes.

### PFT

This test was processed as previously described (Haw et al., 2018). In short, mice underwent anesthesia by intraperitoneal injection with 1% pentobarbital sodium (45 mg/kg) before tracheotomy. Tracheas were cannulated and attached to a Buxco Forced Maneuvers system (obtained from DSI, St. Paul, MN, USA) to assess functional residual capacity (FRC), dynamic compliance (Cydn), resistance index (RI), and minute ventilation (MV) following the manufacturer’s standard testing.

### H&E staining

Lung tissues were perfused with 4% paraformaldehyde (Sigma, Shanghai, China) to prevent alveolar collapse. Then, the lung tissue was fixed with 4% paraformaldehyde for a duration of 24 h, dehydrated in gradient ethanol, embedded in paraffin, sectioned, and stained by means of the H&E staining kit (Beyotime, Shanghai, China). Thereafter, the sections were photographed under a fully automated biological microscope (Leica DM6000B, Leica, Wetzlar, Germany).

### Enzyme-linked immunosorbent assay

The collected patient blood samples were centrifuged at 3,000 rpm for 15 min to separate the serum. Serum KEAP1 and Nrf2 levels were measured using a human KEAP1 enzyme-linked immunosorbent assay (ELISA) kit (EH4240, Wuhan Fine Biotech Co., Ltd., Wuhan, Hubei, China) and a human Nrf2 ELISA kit (abs551899, Absin Bioscience Inc., Shanghai, China) according to the manufacturer’s instructions.

The collected mouse blood samples were centrifuged at 3,000 rpm for 15 min to separate the serum. Serum levels of TG, TC, LDL-C, and HDL-C were measured using the Amplex Red Triglyceride Assay Kit (S0219S), the Amplex Red Cholesterol and Cholesteryl Ester Assay Kit (S0211S), and the Amplex Red High-Density and Low-Density Lipoprotein Cholesterol Assay Kit (S0213S) from Beyotime according to the manufacturer’s instructions.

The collected mouse lung tissues were homogenized to prepare tissue homogenates (10% w/v), and patient serum samples were collected. Changes in oxidative stress levels were assessed using a ROS Assay Kit (ab186029, Abcam, Cambridge, UK), a Human ROS ELISA Kit (mEA-H11876, Shanghai Qiming Biotechnology Co., Ltd., Shanghai, China), and superoxide dismutase (SOD) (A001-3-2), catalase (A007-2-1), and malondialdehyde (MDA) (A003-1-2) assay kits from Nanjing Jiancheng Bioengineering Institute (Nanjing, Jiangsu, China).

### Reverse transcription quantitative polymerase chain reaction

Total RNA was extracted using TRIzol reagent (Invitrogen, Carlsbad, CA, USA). DNA in the cytoplasm and whole cells was isolated using the Genomic DNA Kit (Tiangen, Beijing, China) following the manufacturer’s protocol. qPCR was performed by means of SYBR Premix Ex TaqTM II (Takara, Dalian, Liaoning, China) on the ABI7900HT Fast real-time PCR system (obtained from Applied Biosystems, Foster City, CA, USA). The reaction conditions consisted of 10-min pre-denaturation at a temperature of 95 °C, and 40 cycles of 10-s denaturation at a temperature of 95 °C, 20-s annealing at a temperature of 60 °C, and 34-s extension at a temperature of 72 °C. Data were analyzed by means of the 2^−ΔΔCt^ method. The primer sequences used are depicted in **Table 1**.

**Table 1:** Primer sequences for RT-qPCR.

Gene	Forward 5′–3′	Reverse 5′–3′
KEAP1 (*mouse*)	CGTGGCTGTCCTCAATCGTCT	ATTGCTGTGATCATTCGCCACT
NRF2 (*mouse*)	CAACTCAGCACCTTATATCTCG	ACAAGGAAAACATTGCCATC
NQO1 (*mouse*)	TTCTCTGGCCGATTCAGAG	GGCTGCTTGGAGCAAAATAG
HO-1 (*mouse*)	CACACCCAGGCAGAGAATGCT	GGCTCTCCTTGTTGCGCTCA
GAPDH (*mouse*)	GTCTCC TCT GACTTCAACAGCG	ACCACCCTGTTGCTGTAGCCA A

### Western blotting

Total protein was extracted from cells or lung tissues using radioimmunoprecipitation assay lysis buffer (Beyotime) containing protease inhibitors and phosphatase inhibitors, with concentration quantified by means of BCA Protein Assay Kit (Beyotime). Protein samples were separated by sodium dodecyl sulfate polyacrylamide gel electrophoresis performed in a 4 °C cold room at a stacking gel voltage of 60 V and a separating gel voltage of 120 V for 1–2 h. Following electrophoresis, the proteins were transferred onto polyvinylidene difluoride membranes using the wet transfer method at a constant current of 50 mA per membrane for 2 h in a 4 °C cold room. After blocking with 5% non-fat milk in Tris-Buffered Saline with Tween 20 (TBST) for 1–2 h, the membranes were incubated overnight at 4 °C with primary antibodies. After three TBST washes (10 min each), membranes were incubated with secondary antibody [goat anti-rabbit IgG (1:1,000, ab205718, Abcam) or goat anti-mouse IgG (1:1,000, ab150113, Abcam)] conjugated to horseradish peroxidase for a duration of 1 h at ambient temperature. Following three TBST rinses (10 min each), membranes were subjected to immunoblot visualization using chemiluminescence reagent. Band intensities were quantified using the ImageJ software (National Institutes of Health, Bethesda, MD, USA). β-Actin (1:1,000, ab8226, Abcam) was used as a loading control. The primary antibodies for use included KEAP1 (mouse, 1:1,000, ab119403, Abcam), NRF2 (rabbit, 1:1,000, ab313825, Abcam), NAD(P)H:quinone oxidoreductase (NQO1) (rabbit, 1:1,000, ab80588, Abcam), and heme oxygenase-1 (HO-1) (rabbit, 1:1,000, ab68477, Abcam).

### Statistical analysis

Statistical analyses were performed using SPSS version 27.0 (SPSS Inc., Chicago, IL, USA) and GraphPad Prism version 9.5 (GraphPad Software, San Diego, CA, USA). The normality of continuous variables was assessed using the Shapiro–Wilk test prior to analysis. Data with a normal distribution were presented as mean ± standard deviation, whereas non-normally distributed data were expressed as median (minimum, maximum). For comparisons of continuous variables among multiple groups, one-way analysis of variance (ANOVA) was used when the data were normally distributed, and the homogeneity of variance was confirmed by Levene’s test. When overall statistical significance was observed, Tukey’s multiple comparisons test was applied for post-hoc pairwise comparisons. For data that did not follow a normal distribution, the Kruskal–Wallis test was carried out. Categorical variables were presented as counts (n) and compared using the chi-square test. A correlation analysis was conducted using Spearman’s rank correlation to evaluate the relationships between COPD severity indicators (CAT score and mMRC score) and CAS-related parameters, including the Agatston score, plaque volume, plaque area, and degree of coronary artery stenosis. To further control for potential confounding factors and verify the independence of the observed associations, a multivariable logistic regression analysis was performed for categorical or binary outcome variables. Results were reported as odds ratios (ORs) with corresponding 95% confidence intervals (CIs). All statistical tests were two-sided, and exact *p*-values were reported, with *p* < 0.05 considered statistically significant. The specific statistical methods used for multiple-group comparisons and the notation of statistical significance are indicated in the corresponding figure legends.

## Results

### Comparisons of baseline characteristics

This study retrospectively enrolled 300 consecutive patients with COPD who underwent CTA at Beijing Chao-Yang Hospital from January 2022 to June 2024. Of them, there were 165 COPD cases and 135 cases with COPD + CAS. Additionally, 120 patients with CAS during the same period were recruited as controls. Baseline characteristics of all subjects were collected and compared (**Table 2**). No significant differences were observed in terms of sex, BMI, history of diabetes, history of hypertension, TC, Hb, RBC, and hsCRP among the three groups (all *p* > 0.05). Compared with patients with CAS alone, patients with COPD combined with CAS showed significant differences in age, smoking history, smoking pack-years, CAT score, mMRC score, TG, LPO, GSH-Px, FEV1, FVC, FEV1/FVC, and IL-6 (all *p* < 0.001). Compared with patients with COPD alone, those with COPD combined with CAS also exhibited significant differences in age (*p* = 0.001), smoking history (*p* < 0.001), smoking pack-years (*p* < 0.001), CAT score (*p* < 0.001), mMRC score (*p* < 0.001), TG (*p* < 0.001), LDL-C (*p* = 0.039), HDL-C (*p* = 0.038), LPO (*p* < 0.001), GSH-Px (*p* < 0.001), FEV1 (*p* < 0.001), FVC (*p* < 0.001), FEV1/FVC (*p* = 0.001), and IL-6 (*p* < 0.001). Details are presented in **Table 2**.

**Table 2:** Comparisons of baseline characteristics.

Item	CAS group (n = 120)	COPD group (n = 165)	COPD + CAS group (n = 135)
Age (years)	59.4 ± 10.5	60.8 ± 9.7	64.3 ± 12.3^aa,b^
Sex [female/male, case]	46/74	56/109	50/85
BMI (kg/m^2^)	26.7 ± 3.5	26.7 ± 4.7	27.6 ± 3.7
History of diabetes [n (%)]	30	38	45
History of hypertension [n (%)]	62	83	80
History of hyperlipidemia [n (%)]	70	65	76
Smoking history	68	106	112^aa,bb^
Smoking pack-years	16.00 (0.0, 30.3)	22.0 (0.0, 35.7)	31.1 (0.0, 46.3)^aa,bb^
CAT score	10 (3, 21)	22 (3, 40)	29 (10, 40)^aa,bb^
mMRC score	1 (0, 2)	2 (0, 3)	2 (0, 4)^aa,bb^
TC (mg/dL)	168.1 ± 43.2	151.0 ± 34.7	161.7 ± 40.8
TG (mg/dL)	124.5 ± 30.0	97.4 ± 22.1	110.2 ± 33.7^aa,bb^
LDL-C (mg/dL)	89.9 ± 18.9	81.6 ± 16.0	86.9 ± 19.7^b^
HDL-C (mg/dL)	43.7 ± 14.8	48.4 ± 13.95	44.3 ± 13.5^b^
Hb (g/dL)	14.0 ± 1.9	13.6 ± 1.8	13.8 ± 2.0
RBC (×10^12^/L)	4.3 ± 0.6	4.2 ± 0.5	4.4 ± 0.5
LPO (μmol/L)	6.1 ± 0.9	6.4 ± 1.7	7.2 ± 1.5^aa,bb^
GSH-Px (U/L)	100.8 ± 11.5	102.4 ± 11.8	92.0 ± 10.9^aa,bb^
hsCRP (mg/mL)	8.8 ± 4.8	10.9 ± 3.2	10.1 ± 7.2
FEV1 (% pred)	50.7 ± 8.9	41.9 ± 10.7	32.4 ± 12.6^aa,bb^
FVC (% pred)	68.4 ± 11.5	65.0 ± 13.8	57.2 ± 13.5^aa,bb^
FEV1/FVC	0.8 ± 0.2	0.7 ± 0.1	0.6 ± 0.2^aa,bb^
IL-6 (pg/mL)	32.4 ± 6.2	34.2 ± 6.6	36.1 ± 6.2^aa,bb^

Measurement data consistent with normal distribution were expressed as mean ± standard deviation. Data among multiple groups were compared using one-way ANOVA, followed by Tukey’s post-hoc tests with corrections for multiple comparisons. Data with non-normal distribution confirmed by Kruskal–Wallis test were presented as median (minimum value − maximum value). Categorical variables were compared using chi-square test. COPD, chronic obstructive pulmonary disease; CAS, coronary atherosclerosis; BMI, body mass index; CAT, COPD Assessment Test; mMRC, Modified Medical Research Council Dyspnea Scale; IL-6, interleukin-6; HDL-C, high-density lipoprotein cholesterol; LDL-C, low-density lipoprotein cholesterol; TC, total cholesterol; TG, triglyceride; Hb, hemoglobin; RBC, red blood cells; LPO, lipid peroxidation; GSH-Px, glutathione peroxidase; hsCRP, high-sensitivity C-reactive protein; FEV1, forced expiratory volume in 1 s; FVC, forced vital capacity.

^a^ *p* < 0.05 or ^aa^*p* < 0.01, CAS group *vs*. COPD + CASS group; *p* < 0.05 or ^bb^*p* < 0.01, COPD group *vs*. COPD + CASS group.

### The degree of CAS is correlated with the disease severity in COPD patients

According to the disease severity, COPD patients were allocated into the mild (n = 76), moderate (n = 84), severe (n = 72), and very severe (n = 68) groups. The degree of coronary stenosis in the four groups was compared. It was found (**Table 3**) that in the mild group, 59.21% of patients had coronary arteries with <50% stenosis, 39.47% of patients had 50%–70% stenosis, and 1.32% of patients had more than 70% stenosis. In the moderate group, 46.43% of patients had coronary arteries with <50% stenosis, 44.05% of patients had 50%–70% stenosis, and 9.52% of patients had more than 70% stenosis. In the severe group, 38.89% of patients had coronary arteries with <50% stenosis, 47.22% of patients had 50%–70% stenosis, and 13.89% of patients had more than 70% stenosis. In the very severe group, 41.18% of patients had coronary arteries with <50% stenosis, 38.24% of patients had 50%–70% stenosis, and 22.06% of patients had more than 70% stenosis. The results above show that as the condition of COPD patients worsens, the degree of coronary stenosis also becomes more severe (*p* = 0.004).

**Table 3:** Correlation between the degree of CAS and the severity of COPD in COPD patients.

Degree of coronary artery stenosis	Mild group (n = 76)	Moderate group (n = 84)	Severe group (n = 72)	Very severe group (n = 68)	*P*-value
Mild stenosis (<50%)	45 (59.21%)	39 (46.43%)	28 (38.89%)	28 (41.18%)	0.0041
Moderate stenosis (50%–70%)	30 (39.47%)	37 (44.05%)	34 (47.22%)	26 (38.24%)	
Severe stenosis (>70%)	1 (1.32%)	8 (9.52%)	10 (13.89%)	15 (22.06%)	

Data were compared using chi-square test.

COPD, chronic obstructive pulmonary disease; CAS, coronary atherosclerosis.

### The CAT score or mMRC score in COPD patients is closely tied to CAS

Spearman’s correlation analysis was performed to evaluate the correlations of the CAT score and mMRC score with the Agatston score, plaque volume, plaque area, and coronary stenosis degree in patients with COPD combined with CAS. As shown in **Table 4**, the CAT score was significantly positively correlated with the Agatston score (r = 0.6139), plaque volume (r = 0.8265), plaque area (r = 0.8783), and the degree of coronary artery stenosis (r = 0.7848) (all *p* < 0.01). Similarly, the mMRC score was also significantly positively correlated with the aforementioned clinical parameters (all *p* < 0.01), with correlation coefficients of 0.5015, 0.8182, 0.8502, and 0.7772, respectively. Altogether, these results indicate that the Agatston score, plaque volume, plaque area, and degree of coronary stenosis are significantly related to the CAT score or mMRC score in COPD patients.

**Table 4:** Correlations of the Agatston score, plaque volume, plaque area, and degree of coronary stenosis with the CAT score or mMRC score in COPD patients.

Atherosclerosis indicators	CAT score		mMRC score	
	*r* value	*P*-value	*r* value	*P*-value
Agatston score	0.6139	<0.01	0.5015	<0.01
Plaque volume	0.8265	<0.01	0.8128	<0.01
Plaque area	0.8783	<0.01	0.8502	<0.01
Degree of coronary stenosis	0.7848	<0.01	0.7772	<0.01

Spearman’s test was applied to analyze the correlations of the Agatston score, plaque volume, plaque area, and degree of coronary stenosis with the CAT score or mMRC score in COPD patients.

COPD, chronic obstructive pulmonary disease; CAS, coronary atherosclerosis.

### Oxidative stress response is associated with CAS in COPD patients

**Figure 1:**
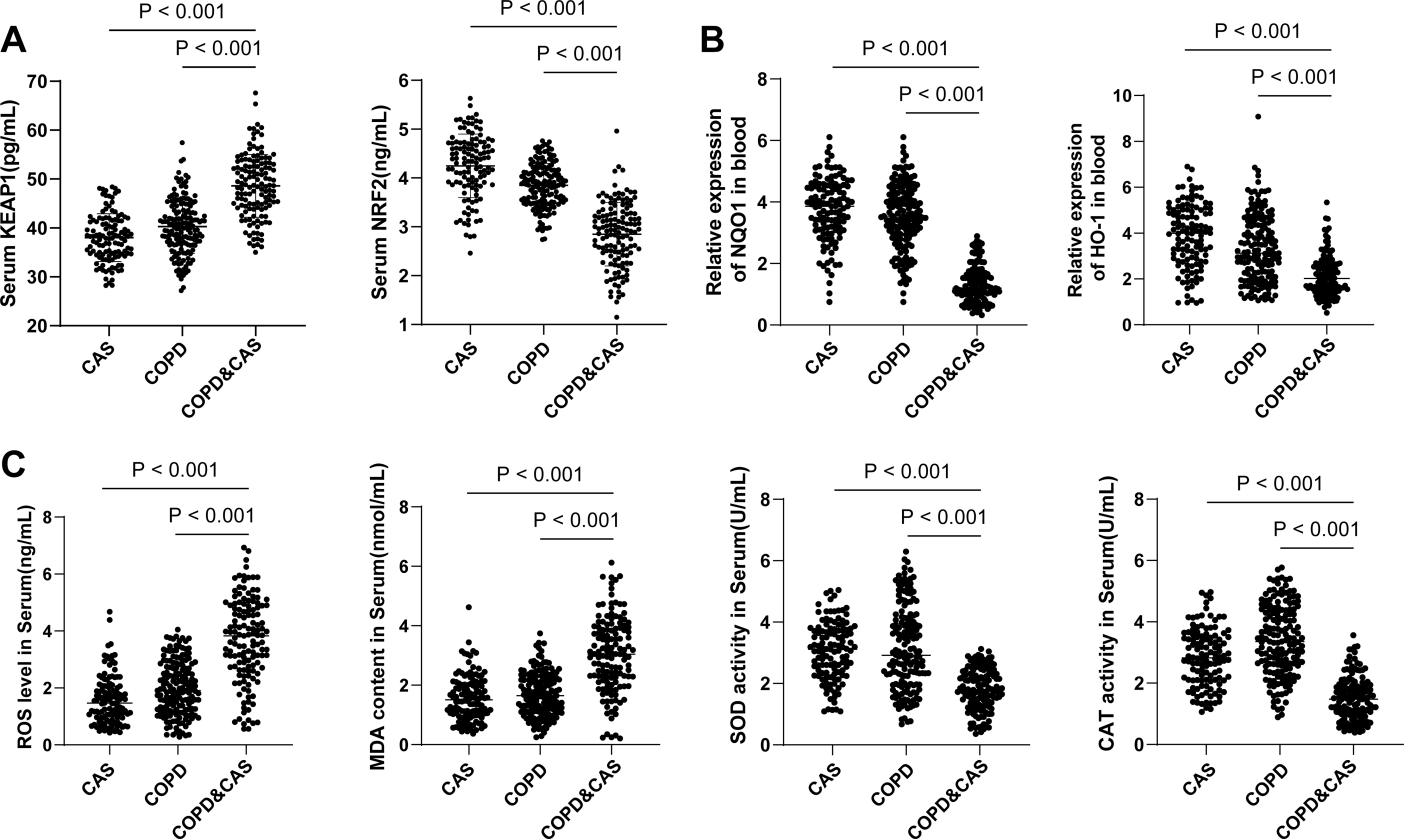
Oxidative stress response and the KEAP1–NRF2 pathway levels are linked to CAS in COPD patients. **(A)** ELISA detection of KEAP1 and NRF2 levels in the serum of patients. **(B)** KEAP1, NRF2, NQO1, and HO-1 levels in the serum of patients determined *via* RT-qPCR. **(C)** Levels of ROS and MDA, and activities of SOD and catalase in the serum of patients determined using kits. The CAS group was used as a normalization control. Data among multiple groups were compared using one-way ANOVA, followed by Tukey’s post-hoc tests with corrections for multiple comparisons. CAS, coronary atherosclerosis; COPD, chronic obstructive pulmonary disease; ROS, reactive oxygen species; MDA, malondialdehyde; SOD, superoxide dismutase.

Serum samples were collected from all patients to detect the changes in the KEAP1–NRF2 pathway and oxidative stress. The results showed higher KEAP1 level and lower NRF2 level in the serum of patients with COPD and CAS than those in COPD patients or CAS patients (all *p* < 0.001) (**Figure 1A**). In addition, compared with COPD patients or CAS patients, the levels of ROS and MDA were increased in the serum of COPD patients with CAS, while the activities of NQO1, HO-1, SOD, and catalase were decreased (all *p* < 0.001) (**Figures 1B, C**). These results established the possible involvement of oxidative stress response and the associated KEAP1–NRF2 pathway in the progression of COPD, and its association with CAS in COPD patients.

### The KEAP1–NRF2 pathway and oxidative stress are linked to the severity of CAS in COPD patients

According to the disease severity, COPD patients were divided into the mild, moderate, severe, and very severe groups. The KEAP1–NRF2 pathway- and oxidative stress-related indicators in each group were compared. As the severity of COPD increased, the levels of KEAP1, ROS, and MDA were upregulated, while the level of NRF2 and the activities of SOD and catalase were decreased (all *p* < 0.001). In addition, COPD patients were assigned to the mild (coronary artery with <50% stenosis), moderate (coronary artery with 50%–70% stenosis), and severe (coronary artery with >70% stenosis) groups according to the degree of CAS. Consistently, as the degree of coronary stenosis increased, KEAP1, ROS, and MDA levels were elevated, while the NRF2 level, and SOD and catalase activities were reduced in COPD patients (all *p* < 0.001) (**Table 5**). Thus, the KEAP1–NRF2 pathway may be involved in COPD progression by mediating oxidative stress and is correlated with CAS severity in COPD patients.

**Table 5:** Analysis on the KEAP1–NRF2 pathway- and oxidative stress-related indicators in COPD patients.

Group allocation basis		KEAP1 (pg/mL)	NRF2 (ng/mL)	ROS (ng/mL)	MDA (nmol/mL)	SOD (U/mL)	Catalase (U/mL)
COPD severity	Mild	35.5 ± 3.0	4.2 ± 0.3	44.1 ± 10.8	15.0 ± 8.3	62.1 ± 10.7	66.7 ± 14.0
	Moderate	41.4 ± 1.9	3.7 ± 0.2	51.5 ± 11.7	16.3 ± 5.3	40.6 ± 6.7	50.9 ± 10.3
	Severe	46.7 ± 1.9	3.2 ± 0.3	60.3 ± 10.5	23.2 ± 6.7	36.5 ± 9.2	32.7 ± 7.2
	Very severe	54.0 ± 3.8	2.4 ± 0.5	74.7 ± 17.9	31.6 ± 6.8	30.1 ± 7.1	28.1 ± 6.8
*P*-value		< 0.001	< 0.001	< 0.001	< 0.001	< 0.001	< 0.001
Degree of coronary stenosis	<50%	37.1 ± 3.7	4.3 ± 0.5	57.6 ± 13.3	19.0 ± 8.0	59.6 ± 11.6	62.3 ± 12.7
	50%–70%	48.3 ± 5.4	3.0 ± 0.5	70.5 ± 11.6	26.4 ± 6.0	47.3 ± 8.5	46.2 ± 10.3
	>70%	53.2 ± 4.9	2.2 ± 0.5	76.9 ± 25.3	33.4 ± 8.5	33.8 ± 9.1	30.9 ± 7.5
*P*-value		<0.001	<0.001	<0.001	<0.001	<0.001	<0.001

Data were expressed as mean ± standard deviation. Data in multiple groups were evaluated using one-way ANOVA, followed by Tukey’s post-hoc tests with corrections for multiple comparisons.

KEAP1, Kelch-like ECH-associated protein 1; NRF2, nuclear factor erythroid 2-related factor 2; COPD, chronic obstructive pulmonary disease.

*p* < 0.05 was considered statistically significant.

### KEAP1 and NRF2 are independent influencing factors of COPD combined with CAS

To further investigate factors influencing the comorbidity of COPD and CAS, a logistic regression analysis was performed with the comorbidity of COPD and CAS as the dependent variable (COPD alone or CAS alone = 0; COPD combined with CAS = 1). Variables showing significant differences in **Table 2**, together with KEAP1 and NRF2, were included in the univariate logistic regression analysis. The results indicated that age, smoking history, smoking pack-years, CAT score, mMRC score, LPO, GSH-Px, FEV1, FVC, FEV1/FVC, IL-6, KEAP1, and NRF2 were associated with COPD combined with CAS. To control for potential confounding factors, variables with *p* < 0.05 in the univariate analysis were subsequently incorporated into a multivariable logistic regression model, while variables with multicollinearity (smoking history, mMRC score, FEV1, and FVC) were excluded. The multivariable analysis indicated that smoking pack-years (*p* < 0.001, OR = 1.068), CAT score (*p* < 0.001, OR = 1.156), LPO (*p* = 0.026, OR = 1.409), GSH-Px (*p* < 0.001, OR = 0.913), KEAP1 (*p* < 0.001, OR = 1.507), and NRF2 (*p* < 0.001, OR = 0.026) were independent influential factors for the comorbidity of COPD and CAS. Details are presented in **Table 6**.

**Table 6:** Logistic regression analyses of factors associated with COPD combined with CAS.

Influencing factors	Univariate analysis	Logistic	Regression	Multivariate analysis	Logistic	Regression
	*P*	OR	95% CI	*P*	OR	95% CI
Age (years)	<0.001	1.035	1.015–1.056	0.081	0.947	0.891–1.007
Smoking history [n (%)]	<0.001	3.106	1.869–5.162	–	–	–
Smoking pack-years	<0.001	1.085	1.062–1.107	<0.001	1.068	1.030–1.108
CAT score	<0.001	1.104	1.077–1.132	<0.001	1.156	1.070–1.169
mMRC score	<0.001	4.802	3.223–7.153	–	–	–
TG (mg/dL)	0.670	1.001	0.995–1.008	–	–	–
LDL-C (mg/dL)	0.359	1.005	0.994–1.017	–	–	–
HDL-C (mg/dL)	0.157	0.989	0.975–1.004	–	–	–
Hb (g/dL)	0.892	1.008	0.904–1.123	–	–	–
LPO (mmol/L)	<0.001	1.552	1.334–1.807	0.026	1.409	1.041–1.907
GSH-Px (mmol/L)	<0.001	0.943	0.925–0.961	<0.001	0.913	0.874–0.954
FEV1 (% pred)	<0.001	0.906	0.886–0.926	–	–	–
FVC (% pred)	<0.001	0.948	0.932–0.965	–	–	–
FEV1/FVC	<0.001	0.023	0.006	0.204	0.152	0.008–1.250
IL-6 (pg/mL)	<0.001	1.069	1.034–1.106	0.287	1.057	0.955–1.169
KEAP1 (pg/mL)	<0.001	1.490	1.377–1.611	<0.001	1.507	1.297–1.751
NRF2 (ng/mL)	<0.001	0.035	0.018–0.069	<0.001	0.026	0.008–0.081

COPD, chronic obstructive pulmonary disease; CAS, coronary atherosclerosis; CAT, COPD Assessment Test; LPO, lipid peroxidation; GSH-Px, glutathione peroxidase; hsCRP, high-sensitivity C-reactive protein; FEV1, forced expiratory volume in 1 s; FVC, forced vital capacity; IL-6, interleukin-6; OR, odds ratio; CI, confidence interval.

### Oxidative stress mediated by the KEAP1–NRF2 pathway is implicated in the occurrence of COPD and CAS comorbidity in mice

**Figure 2:**
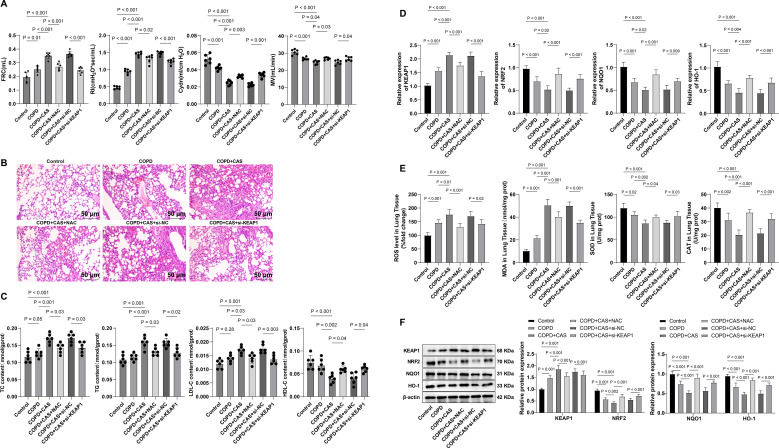
KEAP1–NRF2-mediated oxidative stress participates in the occurrence of COPD combined with CAS in mice. **(A)** PFT for FRC, RI, Cdyn, and MV in mice. **(B)** The histopathological changes of lung tissues observed using H&E staining. **(C)** Serum lipid levels in mice measured *via* ELISA. **(D)** KEAP1, NRF2, NQO1, and HO-1 levels in the serum of mice determined *via* RT-qPCR. **(E)** Levels of ROS and MDA, and activities of SOD and catalase in the lung tissue of mice measured using kits. **(F)** Representative Western blotting of KEAP1, NRF2, NQO1, and HO-1 proteins in the lung tissue of mice and the quantification data. n = 6 mice for each treatment. Data among multiple groups were compared using one-way ANOVA, followed by Tukey’s post-hoc tests for multiple comparisons. COPD, chronic obstructive pulmonary disease; CAS, coronary atherosclerosis; PFT, pulmonary function test; FRC, functional residual capacity; RI, resistance index; Cdyn, dynamic compliance; MV, minute ventilation; ROS, reactive oxygen species; MDA, malondialdehyde; SOD, superoxide dismutase.

Finally, we exposed mice to cigarette smoke to develop a COPD model and constructed a COPD and CAS comorbidity model in combination with a high-fat diet for *in vivo* characterization. PFT results showed that, compared with the control group, the COPD and COPD + CAS groups exhibited significantly increased FRC and RI and significantly decreased Cydn and MV; these alterations were more pronounced in the COPD + CAS group than in the COPD group (all *p* < 0.01) (**Figure 2A**). H&E staining revealed intact and clearly defined alveolar structures in the control group, whereas the COPD and comorbidity groups displayed disrupted alveolar architecture and the presence of emphysematous bullae, accompanied by inflammatory cell infiltration (e.g., macrophages), with more severe pathological changes observed in the COPD + CAS group (**Figure 2B**). The results of lipid profile analysis indicated no significant changes in serum lipids in the COPD group, while the COPD + CAS group showed markedly increased levels of TC, TG, and LDL-C and decreased HDL-C levels (all *p* < 0.01) (**Figure 2C**). Mechanistic analyses suggested that, compared with the control group, KEAP1 expression in lung tissue was significantly upregulated in both the COPD and COPD + CAS groups, whereas NRF2 and its downstream targets NQO1 and HO-1 were significantly downregulated, with more pronounced alterations in the COPD + CAS group (all *p* < 0.01) (**Figures 2D, F**). Consistently, increased ROS and MDA levels and decreased SOD and CAT activities were detected in the COPD group, with more marked changes in the COPD + CAS group (all *p* < 0.01) (**Figure 2E**).

To further verify the pathway mechanism, AAV-si-KEAP1 was administered *via* tail vein injection at week 2 in the comorbidity model. KEAP1 knockdown significantly led to increased levels of NRF2, NQO1, and HO-1 (all *p* < 0.01) (**Figures 2D, F**) and reversed oxidative stress levels (*p* < 0.01) (**Figure 2E**). In addition, compared with the untreated comorbidity group, treatment with the antioxidant NAC or KEAP1 knockdown significantly improved pulmonary function, alleviated lung pathological injury, and ameliorated lipid metabolism disorders in mice (all *p* < 0.01) (**Figures 2A–C**). Taken together, these findings indicate that oxidative stress mediated by the KEAP1–NRF2 pathway plays a critical role in the pathogenesis of COPD combined with CAS, and targeting this pathway may alleviate multisystem injury associated with this comorbidity.

## Discussion

Combining the clinical data and experimental animal data, the current research revealed that oxidative stress mediated by the KEAP1–NRF2 pathway may be a key factor influencing the comorbidity of COPD and CAS. Overall, this study may provide a new strategy for clinical intervention and adjunctive treatment in COPD cases combined with CAS.

In this study, the degree of CAS was observed to be tied to the severity of COPD in COPD patients. As the condition of COPD patients worsened, the degree of coronary stenosis became more severe. Beyond this, strongly positive correlations were noted between atherosclerotic plaque characteristics (including Agatston score, plaque volume, plaque area, and degree of coronary stenosis) and the severity of COPD (CAT score and mMRC score) in COPD patients. CAT is an eight-item self-reported questionnaire widely used to assess the clinical symptoms of patients with COPD, where a higher CAT score indicates greater impact of COPD on the patients’ lives (Bishop et al., 2020). Dyspnea has been stated as the most disabling symptom of COPD; the mMRC scale is a valid and reliable instrument for evaluating the effect of dyspnea on the daily activities of COPD patients, with higher mMRC scores indicative of greater dyspnea severity (Tung et al., 2020; Vilarinho et al., 2022). Moreover, an Agatston score ≥ 1 is indicative of the presence of atherosclerosis (Nunez et al., 2022). Plaque area is a main predictor of CAS, closely associated with disease progression (Spence, 2002). Also, plaque volume and degree of coronary stenosis are important indicators applied to measure the progression of CAS (Hamidi et al., 2024). In support, there is accumulating evidence suggesting that COPD is a risk factor for the development of CAS, independent of other risk factors of cardiovascular events, and the presence of COPD can worsen the clinical prognosis in patients with CAS (Daher and Dreher, 2020).

Oxidative stress has been widely reported to be an important factor linking COPD and CAS, in addition to inflammation, platelet activation, and arterial stiffness (Voulgaris et al., 2021). This stress occurs as a consequence of an imbalance between the production of ROS (including reactive oxygen and nitrogen species, and free radicals) and antioxidants (such as SOD and GSH-Px). Its prolonged presence will result in a decrease in the content of antioxidant status of cells by downregulating the activities of antioxidant enzymes and reductants, amplifying the local inflammatory responses by increasing the gene expression of inflammatory mediators and adhesion molecules, and worsening cardiovascular health, giving rise to COPD-related cardiovascular dysfunctions and mortality (Maclay et al., 2007; Liguori et al., 2018; Hajam et al., 2022; Miklos and Horvath, 2023). Together, these observations strongly support our observation that oxidative stress response was associated with CAS in COPD patients. Accordingly, we strove to investigate the upstream mechanism of oxidative stress and experimentally found *in vivo* that the KEAP1–NRF2 pathway mediated oxidative stress, participated in the COPD progression, and aggravated CAS in COPD. Serum lipid markers TC, TG, and LDL-C showed high levels in a high-fat diet-fed mouse model of atherosclerosis, while HDL-C level exhibited a tendency to decrease (Zhao et al., 2023; Zhou et al., 2023). These levels are correlated with the condition of hypercholesterolemia, a primary risk factor for the occurrence and development of atherosclerosis (Vourakis et al., 2021; Barkas et al., 2023; Liao et al., 2023). The model of ApoE^−/−^ mouse on a high-fat diet is a classical atherosclerosis model that stably produces lipid deposition and plaque burden changes similar to those observed in human CAS, providing a controlled platform for evaluating the impact of COPD-related factors (e.g., smoke exposure and oxidative stress) on atherosclerotic progression (Noonan et al., 2025). In the present study, the mouse model was subjected to cigarette smoke exposure to induce COPD-like lung function decline and pulmonary inflammatory injury (Zhou et al., 2020). Concurrently, under an ApoE^−/−^ background, the mice were fed a high-fat and high-cholesterol diet to accelerate atherosclerotic plaque formation (Gan et al., 2024). This approach enabled the simultaneous manifestation of both a “COPD phenotype” and a “CAS phenotype” in the same animal, thereby mimicking the comorbid condition of COPD and CAS observed in clinical settings. In the COPD and CAS comorbidity mouse model, we found that treatment with antioxidant NAC or KEAP1 knockdown repressed oxidative stress response; improved pulmonary function; alleviated pulmonary pathological changes; diminished TC, TG, and LDL-C levels; and elevated HDL-C levels. As reported, activating the NRF2 signaling is conducive to the enhanced levels of antioxidant genes and decreased levels of oxidative indicators, consequently resulting in protective effects against oxidative stress-induced cell damage and preventing atherosclerosis and related diseases (Zhu et al., 2016; Liao et al., 2023; Jiang et al., 2024). As a major cellular defense mechanism against oxidative stress, NRF2 governs the expression of numerous genes involved in anti-oxidative stress response (Zhao et al., 2021; He et al., 2022). The principal route for NRF2 activity regulation is *via* interaction with its endogenous inhibitor KEAP1 in the cytoplasm. Under normoxia, KEAP1 binds to NRF2 in the cytoplasm, leading to its ubiquitination and degradation; in response to oxidative stress, this interaction is blocked, where NRF2 is dissociated from KEAP1 and then migrates into the nucleus, contributing to a protective role in the prevention of cell damage induced by oxidative stress *via* its regulatory effects on antioxidant genes (Qian and Fan, 2017; Menzel et al., 2021; Ulasov et al., 2022). Moreover, our study analyzed the correlations of KEAP1 and NRF2 with the comorbidity of COPD and CAS in clinical samples. It was revealed that both factors were independent influential factors for the comorbidity of COPD and CAS. The findings of the current study, taken together with those of prior studies, suggest the potential of KEAP1–NRF2-mediated oxidative stress as a predictive biomarker for the diagnosis of CAS or its combination with COPD.

Several limitations of this study should be acknowledged. First, this study was a retrospective, cross-sectional study with samples derived from hospitalized patients who underwent CCTA at a single center. This design is susceptible to selection bias and inherently limits the ability to draw causal inferences. As this was a retrospective study, inter-reader agreement for quantitative statistics [e.g., the intraclass correlation coefficient (ICC)] was not pre-designed for imaging analysis at the initial stage of the study. Second, the absence of a healthy control population restricts data generalizability; the results primarily reflect relative differences among COPD, CAS, and their comorbid state and cannot be extrapolated to the general population. Third, although we adjusted for major imbalanced covariates such as age, smoking history, lipid profiles, and inflammatory markers in multivariable models, residual unmeasured confounders or inadequately controlled confounders cannot be ruled out. Furthermore, due to the lack of detailed pack-year data, we were unable to quantitatively assess the impact of cumulative smoking exposure on the risk of COPD complicated by CAS. Regarding molecular markers, this study measured serum levels of KEAP1/NRF2-related mRNA as circulating biomarkers of systemic oxidative stress, but these markers provide limited insight into tissue-specific sources or cell-specific mechanisms. In the animal experiments, although the combination of a high-fat diet and cigarette smoke exposure in ApoE^−/−^ mice could effectively mimic atherosclerotic pathology in the context of COPD, it remains challenging to completely dissociate the specific effects attributable to COPD versus atherosclerosis. Additionally, the absence of an animal group treated with CAS alone limited further comparisons regarding the isolated effects of atherosclerosis. Future studies with prospective, multicenter designs and more refined animal models are warranted to resolve these limitations.

In summary, the present study identified for the first time the KEAP1–NRF2-mediated oxidative stress as the potential mechanism involved in the comorbidity of COPD and CAS. These results provide a new perspective for the investigation of mechanisms affecting the development of COPD combined with CAS, and also provide a theoretical basis for the development of drugs against COPD combined with CAS by targeting the KEAP1–NRF2 pathway. In the future, more studies remain to be continued, such as a multicenter study with a larger sample size, cell-based experiments for mechanistic verification, and investigations on exact mechanisms related to NRF2 and downstream targets in COPD patients. Additionally, studies with the control of potential confounding variables are warranted. Furthermore, we will further refine the experimental design and incorporate additional experiments to address the limitations of animal modeling.

## Data Availability

The original contributions presented in the study are included in the article/**Supplementary Material**. Further inquiries can be directed to the corresponding author.
