# Supplementary figures and images for: The possible mechanisms linking chronic obstructive pulmonary disease and coronary atherosclerosis based on coronary computed tomography angiography and animal experiments
Source: Front Physiol. 2026 May 7;17:1688832. doi: 10.3389/fphys.2026.1688832 (PMC13189924; doi:10.3389/fphys.2026.1688832)

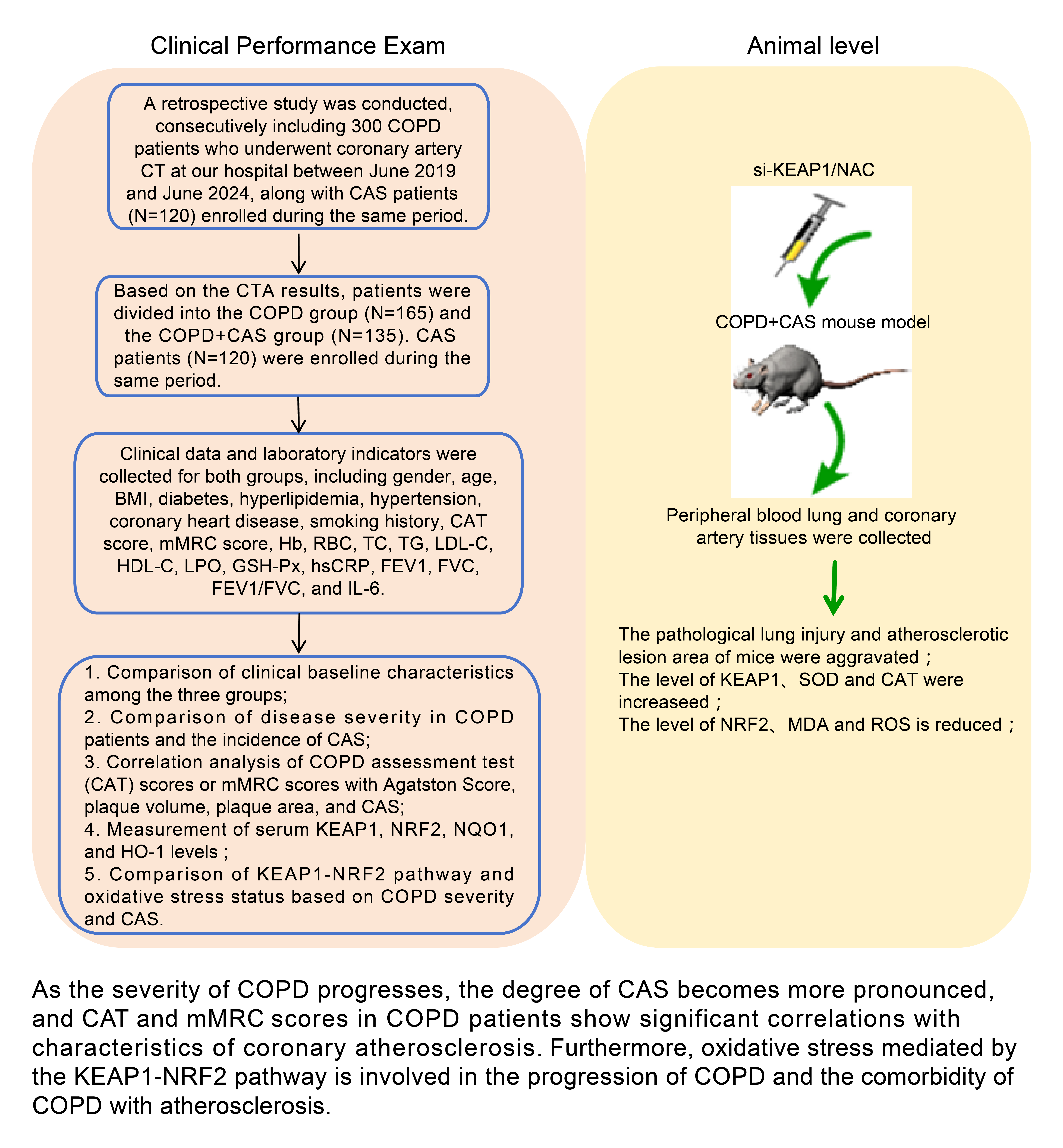

Supplement: Supplementary file 1 [file Image1.tif]
